# Strengthening acute flaccid paralysis surveillance post Ebola virus disease outbreak 2015 - 2017: the Liberia experience

**DOI:** 10.11604/pamj.supp.2019.33.2.16848

**Published:** 2019-05-27

**Authors:** Adolphus Clarke, Nicholas Blidi, Bernice Dahn, Chukwuemeka Agbo, Roland Tuopileyi, Monday Julius Rude, George Sie Williams, Mohammed Seid, Alex Gasasira, Zakari Wambai, Laura Skrip, Thomas Nagbe, Tolbert Nyenswah, Joseph Okeibunor Chukwudi, Ticha Johnson, Ambrose Talisuna, Ali Ahmed Yahaya, Soatiana Rajatonirina, Ibrahima Socé Fall

**Affiliations:** 1 Ministry of Health Monrovia, Monrovia, Liberia; 2 World Health Organization, Country Office, Monrovia, Liberia; 3 National Public Health Institute of Liberia, Monrovia, Liberia; 4 World Health Organization, Regional Office for Africa, Brazzaville, Congo

**Keywords:** Acute Flaccid Paralysis (AFP), surveillance, Liberia, polio

## Abstract

**Introduction:**

Liberia remains at high risk of poliovirus outbreaks due to importation. The country maintained certification level acute flaccid paralysis (AFP) surveillance indicators each year until 2014 due to Ebola outbreak. During this time, there was a significant drop in non-polio AFP rate to (1.2/100,000 population under 15 years) in 2015 from 2.9/100, 000 population in 2013, due to a variety of reasons including suspension on shipment of acute flaccid paralysis stool specimen to the polio regional lab in Abidjan, refocusing of surveillance officers attention solely on Ebola virus disease (EVD) surveillance, inactivation of national polio expert committee (NPEC) and National Certification Committee (NCC). The Ministry of Health (MOH) supported by partners worked to restore AFP surveillance post EVD outbreak and ensure that Liberia maintains its polio free certification.

**Methods:**

We conducted a desk review to summarize key activities conducted to restore acute flaccid paralysis (AFP) surveillance based on World Health Organization (WHO) AFP surveillance guidelines for Africa region. We also reviewed AFP surveillance indicators and introduction of new technologies. Data sources were from program reports, scientific and gray literature, AFP database, auto visual AFP detection and reporting (AVADAR) and ONA Servers. Data analysis was done using Microsoft excel and access spread sheets, ONA software and Geographic Information System (Arc GIS).

**Results:**

AFP surveillance indicators improved with a rebound of non-polio AFP rate (NPAFP) rate from 1.2/100, 000 population under 15 years in 2015 to 4.3 in 2017. The stool adequacy rate at the national level also improved from 79% in 2016 to 82% in 2017, meeting the global target. The percentage of counties meeting the two critical AFP surveillance indicators NPAFP rate and stool adequacy improved from 47% in 2016 to 67% in 2017.The Last polio case reported in Liberia was in late 2010.

**Conclusion:**

There was significant improvement in the key AFP surveillance indicators such as NPAFP rate and stool adequacy with a 3.5 fold increase in NPAFP from 2014 to 2017. By 2017, the stool adequacy rate was up to target levels compared to 2016, which was below target level of 80%. The number of counties meeting target for the two critical AFP surveillance indicators also increased by 20% points between 2016 and 2017. Similarly there was approximately two-fold increase in the oral polio vaccines (OPV) coverage for the reported AFP cases between 2015 and 2017. Strategies employed to address gaps in AFP surveillance included enhanced active case search for AFP, re-instatement of laboratory testing, supportive supervision in addition to facilitating enhanced community engagement in surveillance activities. New technologies such as AVADAR Pilot, electronic integrated supportive supervision (ISS) and electronic surveillance (eSurv) tools were introduced to improve real time AFP case reporting. However, there remain residual gaps in AFP surveillance in the country especially at the sub-national level. Similarly, the newly introduced technologies will require continued funding and capacity building for MOH staff to ensure sustainability of the initiatives.

## Introduction

The global effort to eradicate polio is one of the largest public health initiatives under the global polio eradication initiative (GPEI) launched in 1988 [1]. Polio eradication is also seen as an early win for immunization program in the global vaccine action plan (GVAP) 2011-2020 [2]. The basic strategies for eradicating polio involve, routine immunization, supplemental immunization activities (SIAs), acute flaccid paralysis (AFP) surveillance and house-to-house mopping-up campaigns [1, 3]. The absence of WPV can only be determined by implementation and monitoring of robust AFP surveillance [1, 4]. Maintaining high quality and sensitive AFP surveillance system is important detect any re-emergence or re-introduction of the virus for timely response [1, 3-5]. Polio eradication initiative (PEI) in Liberia started in 2000 and achieved polio free certification 2008 [6]. The country experienced two waves of polio importation in 2009 with 11 WPV1 cases and in 2010 with two cases. However, the country has remained polio free since 2010 [6, 7] and maintained certification level AFP surveillance indicators each year until 2014 due to Ebola outbreak. Liberia has aligned with GPEI polio end game priorities to detect and interrupt all poliovirus transmission, strengthen immunization systems, introduce inactivated polio vaccine (IPV) and withdraw oral polio vaccines (OPV), contain poliovirus and certify interruption of transmission plan polio’s legacy. This paper evaluates the AFP surveillance indicators and describes the results of AFP surveillance post-EVD outbreak in Liberia, demonstrating the rebound in AFP surveillance from 2015-2017 and identifying areas for improvement.

## Methods

**Figure 1:**
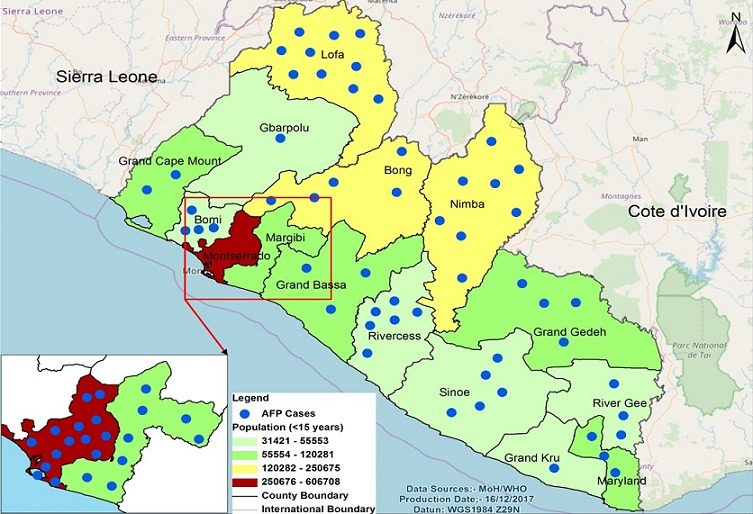
Point map showing number of AFP cases reported by county as of Epi week 52 2017 Liberia

**Study setting:** Liberia is a tropical country in West Africa with a total land area of 111,370 km2. The country is bordered by Sierra Leone in the West, Cote d’Ivoire in the East, Guinea in the North and the Atlantic Ocean in the South. The insert in Figure 1 represents Montserrado County, hosting the nation’s capital city Monrovia. It accounts for one third of the population of Liberia. It is densely populated with several urban slums and high level of population migration. The county dynamics is very diverse and complex. The country has an estimated population of about 4.1 million and an annual growth rate of 2.1%. There are 15 counties (equivalent to WHO districts) and 93 health districts (sub-districts). Based on the National Health Population Census (NHPC), there are more than 200 chiefdoms, 200 clans and 3,694 towns and human settlements within Liberia´s territorial confines national health population commission (NHPC, 2008). Liberia has distinct wet and dry season with the rainy season almost 9 months in the year. The communications and road network as well as the availability of energy sources and distribution is very limited. Accessibility within the country is a great challenge, especially during the rainy season. A total of 96 high priority, 162 medium priority and 546 low priority sites were identified during the period and active case visits conducted by surveillance personnel. The structure for surveillance consisted of the national focal person for Expanded Program on Immunization (EPI) surveillance. He/she worked closely with the county surveillance officers (CSOs) at the first sub-national level. The CSOs, supervise the district surveillance officers (DSOs). The DSOs in turn supervise the focal points at the health facility or community level. Regular meetings are held between staff at the various levels (national, county and community), to facilitate program coordination and implementation activities.

**AFP surveillance process in Liberia:** when a patient meeting the AFP case definition is seen at a health facility or in the community, the district surveillance officers are informed to conduct comprehensive investigations. These include taking a detailed history, conducting a systematic examination, and collecting two stool specimens, 24 to 48 hours apart, within 14 days of onset of symptoms. The stool samples are then transported via DHL to WHO-accredited Regional Reference Polio Laboratory (RRPL) in Abidjan, Cote d’Ivoire for virus isolation and identification. Laboratory results are relayed to the EPI and disease prevention and control (DPC) teams and the surveillance personnel at the sub-national level are required to provide feedback to both the health facilities and community members were the cases were identified. Emphasis was placed on AFP specimen that tested suspected poliovirus and requiring intratypic differentiation. Due to the lapse in AFP indicators during the Ebola outbreak, a series of interventions were implemented to strengthen the system for instance: the verification mechanisms of reported AFP cases at both national and sub-national levels were enhanced. At national and sub-national level, all AFP cases must be reviewed by surveillance personnel, WHO team to verify that the cases meet criteria as true AFP cases, stool specimen collected and sent to the national level. Regular coordination between the national surveillance officer, DPC teams and WHO teams for data sharing and weekly harmonization of surveillance data were conducted. Joint supportive supervision by immunization and surveillance personnel at national and sub-national levels was also conducted. Surveillance officers at county and district level were trained on AFP/ VPD surveillance and additional trainings during IDSR trainings. Clinicians, vaccination teams and general community health volunteers (gCHVs) were sensitized and required to conduct active case search in the communities and during activities such as polio SIAs and african vaccination week (AFW). Regular re-assessment of vulnerability of the surveillance system and prioritization of surveillance sites, identifying high risk areas at county levels and facilitate for prompt and appropriate actions. The use of electronic surveillance tools (ISS, eSurv and AVADAR supervision checklists) enhanced documentation of active surveillance activities. Reinforced AFP surveillance at international cross-borders and port of entry through the integrated diseases surveillance and response (IDSR) framework and during polio campaigns. Counties sharing common borders with Sierra Leone, Guinea and Cote d’Ivoire include Bong, Grand Cape Mount, Grand Gedeh, Nimba, Maryland and Lofa. The country has a national polio expert committee (NPEC) that meets quarterly to conduct final classification of all AFP cases. Liberia presented the annual polio certification updates for 2016 during the Africa Regional Certification Commission (ARCC) annual certification meeting in Malabu, Equatorial Guinea in 2017. The commission accepted the annual update and Liberia maintained her polio free status. An External VPD Surveillance Review in November 2016 supported by WHO and United States Centers for Disease Control and Prevention (CDC) to strengthen quality of VPD surveillance system. Following the review, surveillance improvement work-plan was jointly developed by the MoH and partners and implemented to strengthen the surveillance systems. Additionally, weekly VPD surveillance updates, auto visual AFP detection and reporting sitreps, monthly EPI Bulletin; monthly Brazzaville Initiative (B.I.) reports were produced and disseminated to inform actions.

**Figure 2:**
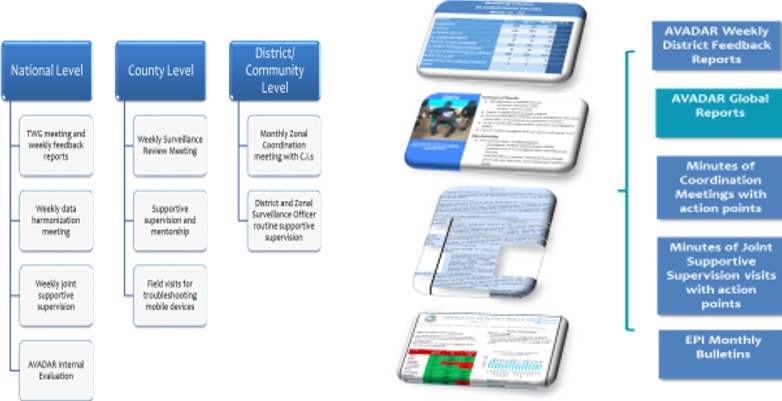
Three level monitoring structure at National, County and District levels

**New technologies:** MOH partnered with WHO, Bill and Melinda Gates Foundation (BMGF), Novel-t and eHealth Africa to pilot auto visual AFP detection and reporting (AVADAR) to enhance community engagement with AFP detection and reporting in Montserrado County. Electronic Integrated Supportive Supervision (ISS) and electronic surveillance (eSurv) checklists were also introduced to enhance active cases search for AFP and other vaccine preventable diseases (VPDs) surveillance through supportive supervision. Within 8 months of introduction, MOH/WHO staff conducted approximately 1,500 supportive supervision visits to health facilities and district teams in all 15 counties using the electronic supervision tools. Three level monitoring structure at National, County and District/ Community; with production and dissemination of information productions for action as shown in Figure 2.

**Data analysis:** Microsoft excel and access ware used data analysis. Data was collected using ODK mobile data application downloaded into smartphones. Data analysis was automatically done for electronic data in the AVADAR and ONA servers and was exported from the servers to excel spreadsheet for further analysis. Arc GIS was used to geo mapping of surveillance and polio campaign activities.

**AFP Surveillance indicators:** the WHO has devised a set of performance indicators to ensure that AFP surveillance is properly maintained. We evaluated the quality of the AFP surveillance using the WHO guidelines for minimum performance standards.

**Ethical considerations:** waiver for ethical approval for the study was obtained from the Liberia Ministry of Health and NPHIL with the confidentiality of patients was maintained during the study.

## Results

During the review period, specimen collection and referral for testing for polio were conducted from all 15 counties. A total of 150 children under 15 years were screened for polio and confirmed negative for wild polio virus from Epi week 1,2016 to Epi week 52 2017. The AFP stool specimens were transported from the point of collection through riders for health or directly by surveillance officers to the national drug store in Monrovia. There the specimens are stored, packaged and transported by DHL Courier to the regional laboratory in Abidjan for testing. Reverse cold chain with appropriate stool storage in the dedicated carries were maintained at all levels. At the same time, 60 day follow-up was conducted for a total of 15 AFP cases and all were reclassified as non-polio cases by national polio expert committees. Re-enforced documentation of 60 day follow-up at both national and sub-national levels.

**Figure 3:**
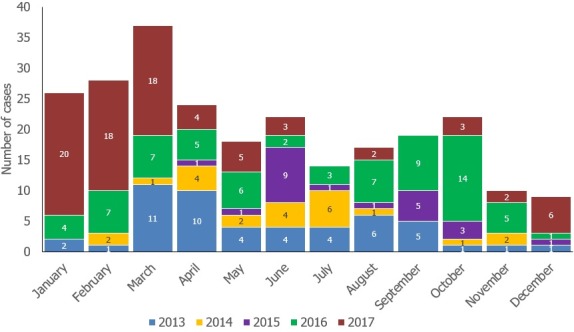
Showing the number of AFP cases reported by month from 2013-2017, Liberia

**Figure 1:** point map showing number of AFP cases reported by county as of Epi week 52 2017 Liberia**Figure 1:** point map showing number of AFP cases reported by county as of Epi week 52 2017 LiberiaThe map in Figure 1 shows the number and distribution by county of AFP cases reported in Liberia as of week 52, 2017. All counties reported at least one AFP case during the review period. Note that the map does not show exact location of the AFP cases as geo coordinates were not captured. However, new technologies such as AVADAR and electronic surveillance tools have the potential to address this gap. Overall there was significant improvement in AFP surveillance indicators during the review period with a rebound of non-polio AFP rate (NPAFP) from 1.2 in 2015 to 3.7 in 2016. As of Epi Week 52, 2017, the NPAFP rate was 4.2. The stool adequacy rate also improved from 79% in 2016 to 82% in 2017, meeting the global target. There was marginal improvement with AFP stool transport time from 47% in 2016 to 50% in 2017 for the reporting period. The % of counties meeting the 2 critical AFP surveillance indicators also improved from 47% in 2016 to 67% in 2017. For more results and other AFP indicators see Table 1 and Table 2, Table 2 (suite). The bar chart in Figure 3 shows that the number of AFP cases reported peaked during the first quarter and plateau for Q2-Q3, with decline between November and December. There was significant increase in the number of AFP cases reported in Q1, 2017 compared previous years. From the table, AFP cases were reported in 100% and 83% of months in 2016 and 2017 respectively.

**Table 1:** AFP surveillance indicators from 2013-2017 (Data Source: MOH/ WHO AFP Surveillance Database)

Indicators	Target	2013	2014	2015	2016	2017
Number of AFP cases Reported		50	23	22	69	81
Non-Polio AFP Rate	>=2/100,000 under 15 Pop.	2.9	1.9	1.2	3.7	4.2
Stool Adequacy Rate	>=80%	100%	96%	95%	79%	80%
Case investigation within 48 hours of report	>=90%	92%	100%	91%	89%	91%
Stool specimens arriving at the lab <3 days of being sent	>=80%	36%	55%	NA	47%	52%
Stool specimens arriving at the laboratory in “good condition”	>=90%	98%	100%	NA	100%	100%
Non Polio enterovirus rate (NPENT)	>=10%	8%	9%	0	12%	23%
% of counties meeting Non- Polio AFP rate and stool adequacy rate	>=80%	80%	60%	53%	47%	67%
60 days follow up	All cases (L)					

**Table 2:** AFP surveillance statistics by county and indicators for 2015 -2016, Liberia (Data Source: MOH/ WHO AFP Surveillance Database)

Year	County	Total AFP cases	Age			Sex		Total polio dases			Durtation from onset-notifi.		Duration from notifi- invest.		Duration from 1st stool -2nd stool		Duration from Onset -2nd stool		2nd stool-national lab		Indicators		
								**dose**			**days**		**days**		**days**		**days**		**days**				
			0-4	5 to 9	>=10	1	2	<3	>=3	unknown	<=7	>7	<=2	> 2	<=2	>2	<=14	>14	<=3	>3	NPAFP	Stool A	NPENT
**2015**	**Bomi**	1	1	0	0	1	0	0	1	0	1	0	0	1	1	0	1	0	0	1	2.3	100%	0%
	**Bong**	0	0	0	0	0	0	0	0	0	0	0	0	0	0	0	0	0	0	0	0	NA	0%
	**Gbarpolu**	1	1	0	0	1	0	1	0	0	1	0	0	1	1	0	1	0	0	1	2.3	100%	0%
	**Grand Bassa**	3	3	0	0	0	3	1	1	1	2	1	3	0	3	0	2	1	0	3	2.6	100%	0%
	**Grand Cape Mount**	0	0	0	0	0	0	0	0	0	0	0	0	0	0	0	0	0	0	0	0	NA	0%
	**Grand gedeh**	2	2	0	0	0	2	0	1	1	2	0	2	0	2	0	2	0	0	2	3.1	100%	0%
	**Grand kru**	1	0	1	0	1	0	0	0	1	1	0	1	0	1	0	1	0	0	1	3.3	100%	0%
	**Lofa**	0	0	0	0	0	0	0	0	0	0	0	0	0	0	0	0	0	0	0	0	NA	0%
	**Margibi**	0	0	0	0	0	0	0	0	0	0	0	0	0	0	0	0	0	0	0	0	NA	0%
	**Maryland**	2	1	0	1	1	1	0	0	2	2	0	2	0	2	0	2	0	0	2	2.8	100%	0%
	**Montserrado**	5	2	3	0	2	3	2	3	0	5	0	5	0	5	0	5	0	0	5	0.9	100%	0%
	**Nimba**	5	3	2	0	1	4	3	2	0	5	0	5	0	5	0	5	0	0	5	2.1	100%	0%
	**Rivercess**	0	0	0	0	0	0	1	0	0	0	0	0	0	0	0	0	0	0	0	0	NA	0%
	**River Gee**	1	1	0	0	1	0	0	0	0	1	0	1	0	1	0	1	0	0	1	2.7	100%	0%
	**Sinoe**	1	0	0	1	1	0	0	0	1	0	1	1	0	1	0	1	0	0	1	1.9	100%	0%

Sex: 1 = female; 2 = male

**Table 2 (suite):** AFP surveillance statistics by county and indicators for 2015 -2016, Liberia (Data Source: MOH/ WHO AFP Surveillance Database)

Year	County	Total AFP cases	Age			Sex		Total polio dases			Durtation from onset-notifi.		Duration from notifi- invest.		Duration from 1st stool -2nd stool		Duration from Onset -2nd stool		2nd stool-national lab		Indicators		
								**dose**			**days**		**days**		**days**		**days**		**days**				
			**0-4**	**5 to 9**	**>=10**	**1**	**2**	**<3**	**>=3**	**unknown**	**<=7**	**>7**	**<=2**	**> 2**	**<=2**	**>2**	**<=14**	**>14**	**<=3**	**>3**	**NPAFP**	**Stool A**	**NPENT**
**2016**	**Bomi**	4	4	0	0	1	3	1	3	0	3	1	3	1	4	0	4	0	3	1	8.9	100%	50%
	**Bong**	11	7	3	1	5	6	2	5	4	4	7	11	0	11	0	6	5	5	6	6.2	55%	18%
	**Gbarpolu**	1	1	0	0	0	1	1	0	0	1	0	1	0	1	0	1	0	0	1	2.3	100%	0%
	**Grand Bassa**	3	3	0	0	2	1	1	0	2	1	2	3	0	3	0	2	1	0	3	2.5	67%	67%
	**Grand Cape Mount**	3	3	0	0	2	1	0	3	0	3	0	1	2	3	0	3	0	2	1	4.4	100%	33%
	**Grand gedeh**	3	1	1	1	2	1	3	0	0	2	1	2	1	3	0	2	1	1	2	4.5	100%	0%
	**Grand kru**	2	2	0	0	0	2	1	1	0	2	0	2	0	2	0	2	0	0	2	6.5	100%	0%
	**Lofa**	7	4	2	1	2	5	2	3	2	7	0	6	1	7	0	7	0	1	6	4.8	100%	0%
	**Margibi**	5	5	0	0	3	2	3	2	0	5	0	4	1	5	0	5	0	2	3	4.5	100%	40%
	**Maryland**	3	2	1	0	3	0	2	1	0	1	2	3	0	3	0	1	2	0	3	2.8	100%	0%
	**Montserrado**	13	8	4	1	6	7	2	6	5	7	6	12	1	13	0	8	5	9	4	2.2	62%	54%
	**Nimba**	7	3	1	3	3	4	0	6	1	4	3	7	0	7	0	5	2	6	1	2.9	71%	14%
	**Rivercess**	1	1	0	0	0	1	0	1	0	1	0	1	0	1	0	1	0	0	1	2.8	100%	0%
	**River Gee**	2	1	1	0	0	2	0	2	0	1	1	2	0	2	0	1	1	1	1	5.3	50%	0%
	**Sinoe**	4	3	1	0	3	1	1	1	2	3	1	4	0	4	0	4	0	0	4	7.4	100%	50%

Sex: 1 = female; 2 = male

**Figure 4:**
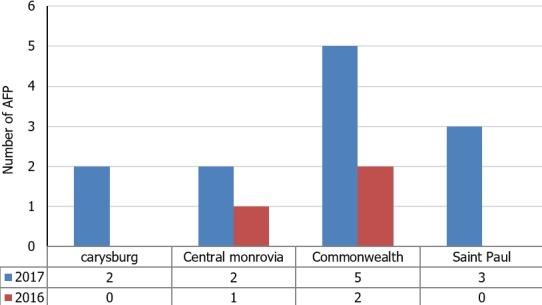
Number of cases reported in AVADAR districts 2017 and 2016

The majority of the reported AFP cases from 2015-2017 were below 5 years of age (n = 112, 65%) and 55% were female. 66% of the AFP cases were notified in less than 7 days from date of symptom onset, with Montserrado, Nimba and Bong Counties accounting for most of the cases notified in more than 7 days from symptom onset. Furthermore, 90% of the AFP cases reported were investigated within 24 hours of notification while the 99% of the 2 stool specimens were collected 24 hours apart. The numbers in Table 2, Table 2 (suite) and Table 3 shows that there was significant improvement in the reported number of OPV doses received by the AFP cases for the period under review. There was approximately a two-fold increase in the number of OPV doses received by AFP cases from 36% in 2015 to 61% in 2017. However, 20% (32/168) of the cases reported unknown number of OPV doses received. In 2015, 0% of AFP stool specimen tested negative for NPENT, while % of NPENT increased from 12% in 2016 to 23% in 2017. Gbarpolu, Maryland, Grand Kru counties did not have any stool specimen positive NPENT between 2015-2017. 52% of stool reached the national level within 72 hours of 2nd stool collection in 2017, an increase from 44% in 2016. Improvement in stool transport times were most evident in Gbarpolu, Grand Bassa, Lofa, Maryland, Rivercess and Sineo Counties, while there was an increase in stool transport times for Nimba, Bomi, Montserrado counties. In Figure 4, it was shown that AVADAR Pilot improved AFP reporting in pilot sites with 12 AFP cases reported between Epi week 11- Epi week 50 2017 compared to 3 AFP cases reported in same pilot sites at same reporting period in 2016. AVADAR Pilot contributed to improve AFP case detection and reporting in previously silent districts as Careysburg district during the period under review. A total of 822 supportive supervision visits were conducted using the eSurv checklist. The checklists were used in all 15 counties. However, there are still areas in some counties were the eSurv checklist have not been used. This can be explained as the eSurv checklist was initially used to 8 counties where Stop the Transmission of Polio (STOP) team were deployed before scaling-up to all 15 counties in Q3, 2017. The aim was to first learn by doing, and subsequently scale up nationwide.

**Table 3:** AFP Surveillance statistics by county and indicators for 2017, Liberia (Data Source: MOH/ WHO AFP Surveillance Database)

Year	County	Total AFP cases	Age			Sex		Total polio dases			Durtation from onset-notifi.		Duration from notifi- invest.		Duration from 1st stool -2nd stool		Duration from Onset -2nd stool		2nd stool-national lab				Indicators
								Doses			Days		Days		Days		Days		Days				
			0-4	5 to 9	>=10	**1**	**2**	**<3**	**>=3**	**unknown**	**<=7**	**>7**	**<=2**	**> 2**	**<=2**	**>2**	**<=14**	**>14**	**<=3**	**>3**	**NPAFP**	**Stool A**	**NPENT**
2017	Bomi	4	3	1	0	1	3	0	4	0	`	2	4	0	4	0	4	0	2	2	8.8	100%	25%
	Bong	5	3	1	1	4	1	0	3	2	5	0	5	0	4	1	5	0	2	3	2.9	100%	0%
	Gbarpolu	2	2	0	0	1	1	1	1	0	1	1	2	0	2	0	1	1	1	1	4.7	50%	0%
	Grand Bassa	4	2	1	1	2	2	1	2	1	2	2	4	0	4	0	3	1	3	1	3.5	50%	50%
	Grand Cape Mount	2	2	0	0		2	0	2	0	1	1	2	0	2	0	2	0	2	1	3.1	100%	0%
	Grand gedeh	4	3	1	0	1	3	1	3	0	4	0	4	0	4	0	4	0	1	3	6.2	100%	50%
	Grand kru	1	1	0	0	1		0	0	1	0	1	1	0	1	0	0	1	0	1	3.4	0%	0%
	Lofa	10	3	5	2	5	5	2	8	0	6	4	6	4	10	0	9	1	5	4	7.1	90%	10%
	Margibi	8	5	2	1	2	6	5	3	0	8	0	8	0	8	0	8	0	5	3	7.5	100%	25%
	Maryland	3	2	1	0	3		2	0	1	0	3	3	0	3	0	2	1	1	2	4.3	67%	0%
	Montserrado	16	12	2	2	7	9	4	11	1	7	9	16	0	16	0	10	6	8	8	2.8	63%	13%
	Nimba	8	5	1	2	4	4	3	5	0	4	4	7	1	8	0	8	0	6	2	3.4	100%	13%
	Rivercess	6	5	1	0	1	5	1	4	1	3	3	5	1	6	0	5	1	3	3	17.6	83%	50%
	River Gee	3	3	0	0		3	0	2	1	1	2	2	1	3	0	1	2	1	2	8.2	33%	67%
	Sinoe	5	4	0	1	2	3	1	1	3	4	1	5	0	5	0	4	1	2	3	9.6	80%	0%

1 = female; 2 = male

## Discussion

As per the desk review, there was significant improvement in the key AFP surveillance indicators between 2014 and 2017 in Liberia such as Non Polio AFP Rate (NPAFP) rate and stool adequacy with a 3.5 fold increase in NPAFP from 2014 to 2017 and with stool adequacy rate reaching global target levels by 2017. The number of counties meeting target for the two critical AFP surveillance indicators also increased by 20% points between 2016 and 2017. Similarly there was approximately two-fold increase in the number of OPV doses received by the reported AFP cases between 2015 and 2017. These improvements with the AFP surveillance indicators can be related to the key activities done in order to improve the capacity and structures of the surveillance system following the EVD outbreak control. For instance, integration of the AFP surveillance within the IDSR framework, improved supportive supervision by surveillance officers and partners, capacity building for surveillance personnel and clinicians, including sensitization and engagement of community health volunteers as well as the high emphasis given for AFP surveillance during Polio NID’s. It also demonstrates the improvement with immunization coverage as more AFP cases were reportedly vaccinated with OPV during the period. From the study, majority of the AFP cases were aged less than 5 years at 65% of total reported cases. This is similar to other studies [8] where 82.5%, 74.3% of the AFP cases below 5 years and demonstrates the most at risk population for AFP and polio cases. Of the total cases reported, 55% were female. AFP case reporting appears to show season variations with more cases reported in Q1 and between September and October. This is similar to other studies [6, 9] showing increased AFP cases reported March and September. This may be associated with the increased capacity to conduct active search for AFP during polio SIAs usually during these months with increased number of health workers and community volunteers deployed for the immunization campaigns. Although during the period under review, AFP cases were reported an average of 90% of the months, heightened AFP case search is required with the same intensity throughout the year to ensure no cases are missed.

Although there were significant rebound of surveillance indicators following restoration of health system recovery, there are still residual gaps in the AFP surveillance with respect to stool transport times and percentage of counties meeting both key AFP surveillance indicators (stool adequacy and NPAFP rate) and percentage of AFP cases receiving three or more doses of Oral Polio Vaccine (OPV) doses at the end of 2017. The residual gap with stool adequacy is associated with the fact only a 66% of the AFP cases were notified in less than seven days from date of symptom onset, thus increasing the chances that collection of stool specimen maybe delayed. This delay in notification of AFP cases within seven days is most pronounced in Montserrado, Nimba and Bong Counties and these counties had inadequate stool specimen during the period under review. However, the study also show that when the AFP cases are notified, response time by the surveillance officers were rapid, for instance 90% of the AFP cases reported were investigated within 24 hours of notification while the 99% of the 2 stool specimen were collected 24 hours apart. This shows that more emphasis should be on active case search for AFP cases and sustained rapid response of the surveillance officers. Some of the contributing factors to residual AFP surveillance gaps include quantity and capacity of surveillance personnel, inadequate surveillance logistics especially for hard to reach areas and sub-optimal community participation for surveillance activities as also highlighted in literature elsewhere [1, 8]. The duration of AFP stool transport time is a critical factor for AFP surveillance as it influences the rate of identification and response for any positive polio case and the quality of stool specimen being tested. Although this paper shows improvement with AFP transport times from 44% in 2016 to 52% in 2017, it still remains below WHO recommended target levels of 80% [5]. From the study, the hard-to-reach counties contributed to the delayed transport times due to poor road access, however some easily accessible counties like Montserrado, Bomi and Bong counties also had delayed AFP transport times. This is very pronounced in the case of Montserrado County which hosts the national depot. Thus there is a need to further review and understand the reasons for long transport times in the county as well as in addressing this gap.

One of the other important AFP surveillance indicators is the Non Polio Entrovirus Rate (NPENT) rate which shows the integrity of stool specimen tested for viral isolation and quality of the reverse cold chain. According to the WHO AFRO region AFP surveillance guideline, the laboratory should be able to isolate the Non-Polio Entero virus in at least from 10% of the total stool samples collected and sent. So, review showed that, there was steady improvement of NPENT rate at the national level from 0% in 2015 to 12% in 2016 and 23% in 2017 [5]. The hard-to-reach counties such as Gbarpolu, Maryland, Grand Kru counties did not have any stool specimen positive NPENT between 2015-2017. As highlighted earlier, the delay in transport time may contribute to the quality of reverse cold chain as it becomes more difficult to maintain as time elapses. Maintaining high level of AFP surveillance is a cornerstone of polio eradication especially for areas with low OPV coverage and risk of polio importation and will require continuous evaluation and assessment of the surveillance system to identify areas of gaps and addressing them [1, 8]. Here the role of operational research for polio eradication remains imperative for AFP surveillance [1, 10]. Liberia immunization program working in the context of IDSR actively uses data to inform strategies to improve and maintain robust AFP surveillance system, for instance in addition to routine M&E systems, the program along with WHO and partners conducted an external VPD surveillance in 2016 to assess the quality surveillance, preparedness and response system [11] in addition to other evaluations. From the study, AFP surveillance system was geographically spread in all 15 counties, including the counties that had lower OPV coverage. However, more efforts should be made to identify all high-risk communities and underserved populations to ensure population immunity and that no polio cases are missed in these areas.

Additionally, key strategies employed to address some of these gaps in AFP surveillance include introduction of new technologies such as AVADAR Pilot, eSurv and ISS electronic surveillance tools which improved the surveillance system. These innovation leverage on technology to strengthen real time AFP case reporting, supportive supervision and accountability mechanism for surveillance personnel. They also facilitate enhanced community engagement in surveillance activities. High level participation and government ownership to introduce the technologies [12] contributed to the success of implementation and contributed to strengthen the surveillance system, demonstrating the capacity of Liberia to adopt and adapt to new technologies. This is especially important as various new technologies such as mobile data collection, GIS, big data and artificial intelligence with capacity to improve health service delivery are being rolled-out simultaneously in developed and developing countries [13-16] and thus Liberia is in a strong position to leverage on technology to improve her immunization program and overall health system. Additionally, these technologies have the capacity to strengthen record keeping and documentation of AFP surveillance activities, as the quality of AFP surveillance is as good as the documented evidence of the activities conducted, as it is the only way to demonstrate the quality of the surveillance system [17]. Furthermore, documentation of AFP surveillance activities and other Polio eradication program (PEP) activities is critical to maintaining polio free certification and archiving of legacy of polio eradication in Liberia [1, 14, 18-20]. On this note, there are various ways in which AFP surveillance can contribute to the polio legacy and continued strengthening of the overall health system [18, 19]. For instance, the PEP personnel at both national and sub-national level contributing to build capacity of district teams and health workers on immunization service delivery and VPD surveillance. The logistic supplies for AFP surveillance also support surveillance for other VPDs and integrated supportive supervision for immunization activities [21-23]. Furthermore, the introduction of new technologies is already being leveraged by other programs especially the IDSR team to strengthen real time data collection, supportive supervision, feedback systems and accountability systems for staff in Liberia.

## Conclusion

During the period under review, Liberia demonstrated strong rebound in AFP surveillance indicators as the country restored essential health service delivery following significant decline due to the devastating EVD outbreak in 2014-2015. These improvements were most significant in the two critical AFP surveillance indicators at the national level, thus ensuring high level vigilance for AFP cases in line with polio end game activities. These improvements were due to significant human and material resources deployed to rejuvenate the surveillance systems post EVD within investment plan for rebuilding a resilient health system. However, more needs to be done to sustain the gains made thus and further improve the system especially at the sub-national level. Here, some counties perform sub-optimally in some AFP surveillance indicators predisposing to risk of missing any polio case. On this note, Liberia is working closely with partners to pilot and deploy innovations to strengthen surveillance systems especially in areas that are at high risk to polio importations.

### What is known about this topic

- Devastating impact of Ebola outbreak on health systems and performance indicators in affected countries;
- Use of AFP surveillance system for early case identification of VPDs and Ebola virus in different countries;
- Use of technology to strengthen AFP surveillance and other polio eradication activities in other countries.

### What this study adds

- Experience of Liberia in reviving AFP surveillance indicators following Ebola outbreak;
- Strategies deployed to improve AFP surveillance indicators in Liberia;
- Presence of residual gaps which needs improvement to maintain Polio certification in Liberia.

## Competing interests

The authors declare no competing interest.
